# Access and Use of Services by Caregivers of Older Adults: A Scoping Review of Cultural and Linguistic Diversity

**DOI:** 10.1177/07334648231158490

**Published:** 2023-03-03

**Authors:** Danielle Knipping, Anna Garnett, Bingfang Bianca Jiang

**Affiliations:** 1 Nursing, 6221 University of Western Ontario, London, ON, Canada

**Keywords:** home- and community-based care and services, caregiving, diversity and ethnicity, access to care

## Abstract

Westernized countries are home to an increasingly culturally and linguistically diverse (CLD) older adult population. Informal caregivers of CLD older adults face unique challenges accessing and using home- and community-based services (HCBS). This scoping review sought to identify facilitators and barriers to access and use of HCBS for informal caregivers of CLD older adults. Arksey and O’Malley’s framework guided a systematic search of five electronic databases. The search strategy retrieved 5979 unique articles. Forty-two studies met the inclusion criteria and informed this review. Facilitators and barriers were identified at three stages of using services: knowledge, access, and use of services. Findings concerning access to HCBS were subdivided into willingness and ability to access HCBS. Results emphasize the need for changes in healthcare systems, organizations, and providers to provide culturally appropriate care and improve the accessibility and acceptability of HCBS for informal caregivers of CLD older adults.

**What this paper adds**

• This paper is the first review to examine facilitators and barriers to HCBS access and use for CLD caregivers of older adults in Westernized countries.
• This paper includes diverse perspectives from caregivers with various cultural and linguistic identities living in seven countries.
• This paper identifies unique facilitators to access and use of HCBS including family and friends, immigrant-serving organizations, and trusting relationships with healthcare providers.

**Applications of study findings**

• Review findings identify support with system navigation and language barriers as high priorities for overcoming barriers to access and use of HCBS for CLD caregivers.
• Review findings support changes to systems and organizations including promoting diversity in the healthcare field, promoting collaboration between mainstream and culturally tailored services, and developing policies that meet the needs of diverse populations.
• Review findings highlight several areas requiring further research to better support the needs of CLD caregivers in culturally appropriate ways, such as exploring more diverse caregiving perspectives, disseminating information about HCBS, and understanding facilitators to accessing HCBS.

Westernized countries are increasingly composed of a culturally and linguistically diverse (CLD) older adult population due to global aging and migration trends (Khan, 2019). Older adults are more likely to have greater care needs than other age groups, usually related to multiple chronic conditions such as dementia or arthritis that impact activities of daily living (Khan, 2019). These needs are often fully or partially met by informal caregivers, typically close family members who provide unpaid support including transportation, personal and medical care, housework, financial management, and emotional support (Adelman et al., 2014; Khan, 2019). While the informal caregiving role can be fulfilling, it can also negatively impact caregivers’ mental and physical health, finances, and employment (Adelman et al., 2014; Pristavec, 2019). Informal caregiving can be particularly challenging for caregivers who are older adults themselves or who are juggling employment and/or care for children (Pristavec, 2019). Home- and community-based services (HCBS) support the caregiver, care recipient, or both (Anngela-Cole & Hilton, 2009) and can improve quality of life and support older adults to age in place (Greenwood et al., 2015).

While informal caregiving experiences are well documented, most research in Westernized countries focuses on White caregiver experiences (Robinson et al., 2013; Verbakel et al., 2018). This research often does not consider CLD communities’ diverse beliefs, values, and experiences that impact informal caregivers’ perception of their role and its stressors (Johl et al., 2016). This review defines CLD as having a culture or language that differs meaningfully from the host country’s majority population, often related to migration, Indigenous status, and/or religion (Marcus et al., 2022).

HCBS in Westernized countries are not always accessible to CLD caregivers and do not always meet their needs and may therefore increase CLD caregivers’ stress (Anngela-Cole & Hilton, 2009; Greenwood et al., 2015). While caregivers of all backgrounds have low uptake of HCBS, the literature indicates that CLD caregivers underuse services compared to caregivers from majority populations (Greenwood et al., 2015). This discrepancy has been attributed to a range of complex and diverse factors including systemic disadvantages, community and family beliefs, language proficiency, and socioeconomic status (Greenwood et al., 2015).

The increasingly diverse population of older adults in Westernized countries coupled with lower uptake of HCBS by CLD caregivers (Greenwood et al., 2015; Khan, 2019) point to a need to better understand the facilitators and barriers CLD informal caregivers face in accessing and using HCBS to meet the needs of diverse populations. To our knowledge, this is the first review focused on the experiences of informal caregivers of CLD older adults in Westernized countries with HCBS that is not limited by the care recipient’s diagnosis. Existing literature reviews on CLD caregiver use of HCBS focus on caregivers of persons with dementia (Duran-Kiraç et al., 2021; Johl et al., 2016; Kenning et al., 2017) or a wide range of care recipient ages (Greenwood et al., 2015). Therefore, the aim of this scoping review was to review the current literature to identify facilitators and barriers to the access and use of HCBS by informal caregivers of CLD older adults living in Westernized countries.

## Methods

A scoping review methodology was chosen due to its strengths in summarizing and disseminating findings on a broad topic (Arksey & O’Malley, 2005). The review followed the five-stage framework outlined by Arksey and O’Malley (2005) and the Preferred Reporting Items for Systematic reviews and Meta-Analyses extension for Scoping Reviews (PRIMSA-ScR; see Supplementary File 1; Tricco et al., 2018). The review protocol was not registered or published in peer-reviewed publications.

### Stage 1: Identify the Research Question

This review sought to answer the following research question: What are the facilitators and barriers to access and use of HCBS for informal caregivers of CLD older adults living in Westernized countries?

### Stage 2: Identify Relevant Literature

An academic librarian assisted in developing the search strategy, which was executed by BJ and DK in five databases (MEDLINE [Ovid], Embase [Ovid], CINAHL, PsycINFO [Ovid], and Web of Science) and was limited to literature published from January 2010 until October 12, 2022, the date of the final search, to capture the current state of the literature on this topic (see Supplementary File 2). Search results were imported into *Covidence* online software (https://www.covidence.org) by BJ and DK. The search strategy was limited to database searches due to time constraints.

### Stage 3: Study Selection

At least two authors (AG, BJ, and DK) independently screened records by title and abstract, then by full text using *Covidence*. Disagreements were resolved with all authors. The inclusion criteria required included articles to be full-text, primary studies, English-language, and published in a peer-reviewed journal; have a study sample that included informal caregivers of community-dwelling CLD older adults in a Westernized country; and report caregivers’ facilitators and/or barriers to access or use of HCBS. Westernized countries were defined as European Union members plus Australia, Canada, Iceland, New Zealand, Norway, Switzerland, the United Kingdom, and the United States. Older adults were defined as having an average age over 65, having a diagnosis typically occurring in older adults, for example, dementia, or those identified as older adults, seniors, or elderly by study authors. Studies were excluded if informal caregivers did not live in the same geographical region as care recipients such that they could not provide regular in-person care, or caregiver perspectives were not included in the findings. One author (DK) performed a critical appraisal of the included studies using the Joanna Briggs Institute critical appraisal tools to enhance the quality of the review (Lockwood et al., 2015; Moola et al., 2020). Articles were not excluded based on quality since this is not required within the Arksey and O’Malley (2005) framework.

### Stage 4: Charting the Data

At least two researchers (BJ, CO, and DK) independently extracted data pertaining to host country, participants’ CLD identity, study design, study aim, sample size, facilitators and barriers to HCBS access and use related to CLD identity, and funding source.

### Stage 5: Collating, Summarizing, and Reporting Results

Review results are presented through descriptive statistics of included studies and a narrative summary of findings. Implications of the analysis are then discussed.

## Results

**Figure 1.**
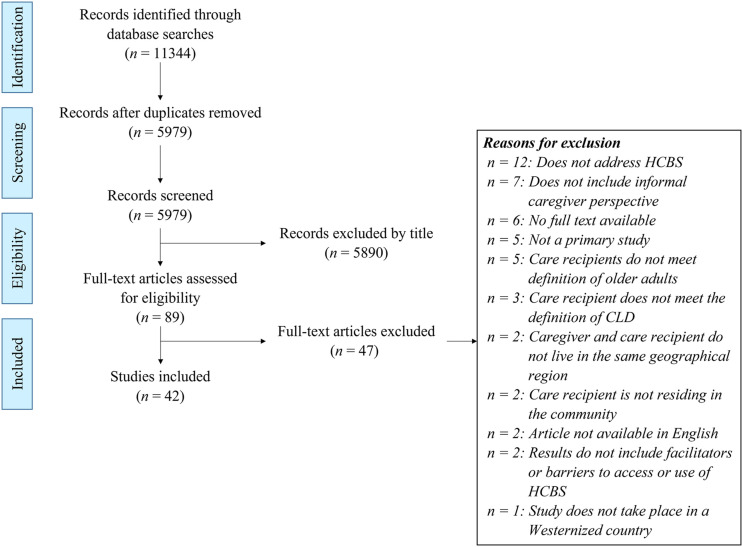
PRISMA-ScR diagram.

The systematic search yielded 5979 unique citations after duplicates were removed. Screening by title and abstract excluded 5890 articles, and full-text reviews excluded 47 additional articles (see Supplementary File 3). The remaining 42 articles met the review inclusion and exclusion criteria and were included in the final analysis. Study selection was reported according to PRISMA-ScR guidelines (see Figure 1; Tricco et al., 2018). The included studies took place in nine countries and included participants representing 44 cultural or linguistic identities, although some identities overlapped, for example, South Asian and Indian (see Table 1). Some studies (*n* = 25) focused on caregivers of care recipients with dementia, heart disease, or stroke. The critical appraisal for studies using qualitative methods yielded an average score of 5.6/10, and the one study that was appraised for cross-sectional methods scored 6/7 (see Supplementary File 4).

**Table 1.** Characteristics of Included Studies.

Citation	Country	CLD identity	Aim of study	Study design	Sample size^a^	Funding source
Armstrong et al. (2022)	UK	Black, South Asian	Explore caregivers’ experiences of dementia care and the impact of COVID-19	Exploratory qualitative	11	NIHR School for Primary Care Research
Arora et al. (2020)	Norway	Pakistani	Explore female caregivers’ views on the future of formal and informal care of older relatives	Qualitative	10	Not stated
Baghirathan et al. (2020)	UK	African-Caribbean, South Asian, Chinese	Generate a grounded theory informed by the experiences of caregivers and their reluctance to access dementia services	Grounded theory	27	Bristol City Council’s Public Health Team
Benedetti et al. (2013)	Australia	Italian	Explore the experiences of caregivers caring for a loved one with dementia	Phenomenology	9	Not stated
Berdai Chaouni and De Donder (2019)	Belgium	Moroccan	Understand how informal caregivers experience dementia and the care of elders, how culture and religion influence experiences	Qualitative	12	No financial support
Berdai Chaouni et al. (2020)	Belgium	Moroccan	Understand the experiences of informal caregivers of older migrants with dementia	Cross-sectional qualitative	12	Not stated
Biswas et al. (2022)	Canada	Foreign-born	To identify the factors that may influence access to dementia care and support programs	Interpretive qualitative	3	No financial support
Blix and Munkejord (2022)	Norway	Indigenous Sami	Explore experiences of barriers to accessing public care services for older adults living with dementia	Interpretive descriptive	11	Norwegian Research Council
Browne and Braun (2017)	US	Native Hawaiian	Explore perspectives on aging, caregiving, and care preferences	Qualitative	30^b^	Administration on Community Living, DHHS
Browne et al. (2014)	US	Native Hawaiian	Investigate health needs and care preferences	Listening study format	17	US Administration on Aging, DHHS
Casado and Lee (2012)	US	Korean	Examine access barriers and identify predictors of unmet needs for HCBS	Cross-sectional survey	146	Not stated
Casado et al. (2015)	US	Korean	Understand the experiences of family caregivers of PWD	Qualitative	23	Not stated
Cooper et al. (2020)	Canada	Métis	Examine the experiences of caregivers providing care for older adults	Qualitative	79	Canadian Institutes of Health Information
Crist and Speaks (2011)	US	Mexican	Explore cultural norms that contribute to decisions of older adults and their caregivers on use of homecare services	Ethnography	3	Not stated
Czapka and Sagbakken (2020)	Norway	Somali, Polish, Pakistani, Indian, Croatian, Turkish^c^	Identify barriers and facilitators in accessing and using dementia care experienced by CLD caregivers	Qualitative	8	Stiftelsen Dam (Extrastiftelsen)
Farrar et al. (2018)	US	Amish	Describe and explore the perspectives of caregivers of older adults and their experiences with health care services outside of their community	Phenomenology, CBPA	15	D. W. Reynolds Foundation of Geriatric Nursing Excellence; Jonas Foundation
Gelman (2010)	US	Puerto Rican, Cuban, Dominican, Ecuadorian, Colombian	Understand the experience of family caregivers caring for a loved one with Alzheimer’s disease	Categorical-content narrative analysis	10	New York University Alzheimer’s Disease Center (NIH NIA)
Giuntoli and Cattan (2012)	UK	Pakistani, Ukrainian, Afro-Caribbean, Indian, Italian, Bangladeshi, Polish, Hungarian	Explore the extent to which older migrants’ expectations and use of services differ from those of national majorities and their relevance to culturally sensitive practices	Narrative approach	33	Joseph Rowntree Foundation
Greenwood et al., 2016	UK	Pakistani, Indian, Black Caribbean, Black African	Explore the experiences of caregivers of stroke survivors in accessing and receiving social care services	Qualitative, in-depth approach	41	NIHR SSCR
Habjan et al. (2012)	Canada	First Nations	Understand perspectives and experiences of obtaining health services and social support for elderly people	Mixed methods^d^	70	Not stated
Haralambous et al. (2014)	Australia	Chinese, Vietnamese	Determine barriers and enablers to accessing dementia services for PWD and their families	Qualitative	13	J. O. & J. R. Wicking Trust
Herat-Gunaratne et al. (2020)	UK	Bangladeshi, Indian	Explore how caregivers’ cultural identities and values influence their experiences and their relationship with services	Qualitative	10	Alzheimer’s Society (UK)
Horsfall et al. (2016)	Australia	Greek	Understand how caregivers make decisions to accept or reject HCBS	Longitudinal qualitative	13	University of Western Sydney; SGMRC; SESLHD; UNSW
Jung Kim et al. (2019)	US	Korean	Explore the experiences of caregivers of PWD and understand how they try to fill unmet service needs	Qualitative	18	Arthur N. Rupe Foundation; NIH NCRR; NIH NCATS
Ketchum et al. (2022)	US, Germany	Black or African American, Hispanic or Latino, Turkish	To identify barriers and facilitators to the use of formal dementia services	Descriptive qualitative approach	18	UW-Madison School of Medicine & Public Health; DZNE; NIH NIA; NIH NIMHD
Koehn et al. (2019)	Canada	Korean, Punjabi	Explore the role of immigrant-serving agencies in facilitating access for caregivers to dementia services/supports	Critical interpretive approach	15	Alzheimer’s society Research Program, Canada
Krishnamurthi et al., 2022	New Zealand	Indian	Explore the lived experiences of PWD and their caregivers	Qualitative	10	The University of Auckland
Lee and Smith (2012)	US	Korean	Gain insight into caregiving experiences of caregivers for PWD	Phenomenology and case study	8	Not stated
Liu and McDaniel (2015)	Canada	Chinese	Examine service gaps, needs, and barriers for people with heart disease/stroke, family caregivers, and health providers	Qualitative	20	Chinese Canadian Council of the Heart and Stroke Foundation
Martinez et al. (2022)	US	Latino	Highlight the experience of care for family caregivers to PWD and their preferences for supportive services	Qualitative	24	NIH NIA
Martinez & Acosta Gonzalez (2022)	US	Latino	Explore the cultural scripts and gendered practice of care in relation to the underutilization of services to PWD	Qualitative	24	NIH NIA
Motta-Ochoa et al. (2021)	Canada	Jewish, Caribbean, Italian	Explore intersections of culture and social inclusion/exclusion in PWD, caregivers, and non-profit organization staff	Ethnographic approach	9^e^	No financial support
Nielsen et al. (2020)	Denmark	Turkish, Pakistani, Arabic	Explore barriers in access to dementia care in CLD communities	Hermeneutic phenomenology	12	The Velux Foundations; the Danish Ministry of Health
Nkimbeng et al., 2022	US	African immigrants	Identify dementia care needs and assets of the community	Exploratory mixed-methods design^f^	24^g^	UMN PHDR; UMN School of Public Health; NIH NCI
Pound and Greenwood (2016)	UK	Indian, Pakistani, Black African, Black Caribbean	Explore post-stroke experiences of older caregivers receiving homecare to help services be responsive to their needs in culturally sensitive ways	Phenomenology	50	NIHR SSCR
Richardson et al. (2019)	US	African American, Hispanic, South Korean	Explore cultural factors underlying caregivers’ experiences and use of services	Ethnocultural	15	Ohio State University College of Social Work; Coca-Cola Critical Difference for Women grant
Shanley et al., 2012	Australia	Chinese, Spanish, Italian, Arabic	Understand what dementia HCBS CLD communities use, issues faced in using HCBS, and how to improve HCBS	Qualitative	121	Australian National Health and Medical Research Council
Strudwick and Morris (2010)	UK	African-Caribbean	Explore perspectives of informal stroke caregivers	Qualitative	9	Not stated
Sun et al. (2014)	US	Chinese	Explore perceptions of service barriers	Qualitative	6	Not stated
Williams et al. (2021)	US	American Samoan	Explore how cultural norms influence caregivers’ knowledge, attitudes, and care choices for PWD	Mixed methods^d^	20	ANA and Health Resources & Services Administration
Willis et al. (2016)	UK	South Asian	Explore experiences and satisfaction with social services	Qualitative	36^e^	NIHR SSCR
Xiao et al. (2015)	Australia	Vietnamese	Explore the perceived challenges of dementia care	Philosophical hermeneutic	6	Faculty of Health Sciences, Flinders University

*Note.* Administration for Native Americans (ANA). Community-based participatory approach (CBPA). Department of Health and Human Services (DHHS). German Center for Neurodegenerative Diseases (DZNE). National Center for Advancing Translational Sciences (NCATS). National Cancer Institute (NCI). National Center for Research Resources (NCRR). National Institute on Aging (NIA). National Institutes of Health (NIH). National Institute for Health Research (NIHR). National Institute on Minority Health and Health Disparities (NIMHD). Program in Health Disparities Research (PHDR). Persons with dementia (PWD). South Eastern Sydney Local Health District (SESLHD). St George Migrant Resource Center (SGMRC). School of Social Care Research (SSCR). United Kingdom (UK). University of Minnesota (UMN). University of New South Wales (UNSW). United States (US). University of Wisconsin (UW).

^a^Indicates the sample of participants who meet inclusion/exclusion criteria for study unless otherwise noted.

^b^Includes elders and key informants.

^c^Includes one participant from an unspecified island in the Atlantic Ocean.

^d^Quantitative results presented in the publication do not meet inclusion/exclusion criteria and therefore are not included in the data analysis.

^e^Includes caregivers from a non-CLD population.

^f^Quantitative findings not reported in the publication.

^g^Includes transnational caregivers.

**Figure 2.**
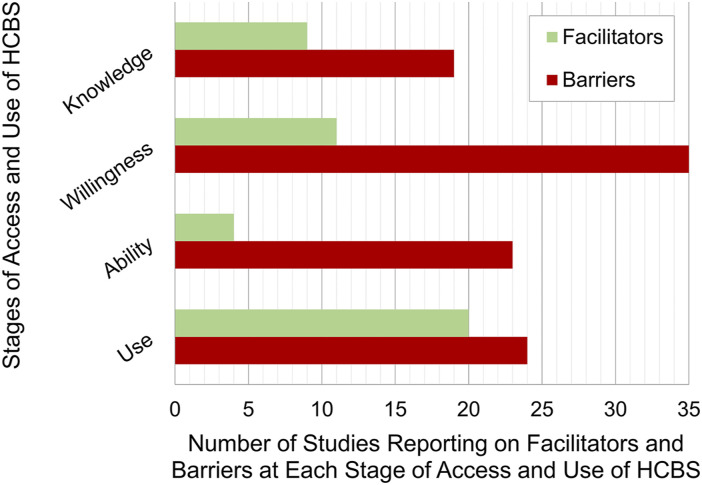
Proportional representation of citations referencing the temporal stages when facilitators and barriers are experienced by CLD caregivers using services.

The scoping review results are presented according to three temporal stages of HCBS use addressed throughout the included studies: (a) acquiring knowledge about HCBS (*n* = 20), (b) accessing HCBS (*n* = 42), and (c) using HCBS (*n* = 32). Findings concerning caregivers’ access to HCBS are subdivided into (i) willingness to use HCBS (*n* = 36) and (ii) ability to access HCBS (*n* = 24) (see Figure 2).

### Knowledge of Services

Caregivers needed to know about HCBS to access and use HCBS. Culturally appropriate facilitators to knowledge were healthcare providers who understood the barriers they faced (Czapka & Sagbakken, 2020; Ketchum et al., 2022) and immigrant-serving community organizations (Browne & Braun, 2017; Koehn et al., 2019; Xiao et al., 2015). Caregivers used persistence, desperation, informal networks, and luck to access information if knowledge was not accessible through formal avenues (Berdai Chaouni & De Donder, 2019; Berdai Chaouni et al., 2020; Czapka & Sagbakken, 2020; Gelman, 2010; Nielsen et al., 2020). Barriers to knowledge identified in the included studies were difficulty navigating healthcare systems (Berdai Chaouni et al., 2020; Berdai Chaouni & De Donder, 2019; Blix & Munkejord, 2022; Browne et al., 2014; Browne & Braun, 2017; Casado & Lee, 2012; Czapka & Sagbakken, 2020; Gelman, 2010; Haralambous et al., 2014; Nielsen et al., 2020; Pound & Greenwood, 2016; Shanley et al., 2012; Sun et al., 2014; Willis et al., 2016; Xiao et al., 2015) and difficulty understanding information about HCBS (Czapka & Sagbakken, 2020; Haralambous et al., 2014; Nielsen et al., 2020; Willis et al., 2016). Studies focusing on caregivers of persons with dementia reported delayed dementia diagnosis as a barrier to subsequent access to knowledge about dementia-specific services (Baghirathan et al., 2020; Biswas et al., 2022; Gelman, 2010; Ketchum et al., 2022; Krishnamurthi et al., 2022; Nielsen et al., 2020; Shanley et al., 2012).

Caregivers in included studies accessed knowledge about HCBS from a variety of sources. Knowledge came from healthcare providers who shared their CLD identity or understood the barriers they faced (Czapka & Sagbakken, 2020; Ketchum et al., 2022), or immigrant-serving agencies or community organizations that disseminated information about HCBS (Browne & Braun, 2017; Koehn et al., 2019; Xiao et al., 2015). These organizations needed close communication with HCBS organizations to ensure their information was complete and accurate (Koehn et al., 2019; Xiao et al., 2015). In the absence of accessible formal sources of knowledge, desperation, informal networks, persistence, and luck facilitated access information, for example, friends or family with insider knowledge of HCBS (Berdai Chaouni & De Donder, 2019; Berdai Chaouni et al., 2020; Czapka & Sagbakken, 2020; Gelman, 2010; Nielsen et al., 2020).

Conversely, many caregivers in included studies struggled with the complexity of healthcare systems, reporting that they did not always know what services were available or how to access information (Berdai Chaouni et al., 2020; Berdai Chaouni & De Donder, 2019; Blix & Munkejord, 2022; Browne et al., 2014; Browne & Braun, 2017; Casado & Lee, 2012; Czapka & Sagbakken, 2020; Gelman, 2010; Haralambous et al., 2014; Nielsen et al., 2020; Pound & Greenwood, 2016; Shanley et al., 2012; Sun et al., 2014; Willis et al., 2016; Xiao et al., 2015). Caregivers from countries without comparable services may have limited expectations of HCBS in their new country (Shanley et al., 2012). Some caregivers reported that they could not always understand the information they received about HCBS due to language barriers or literacy skills (Czapka & Sagbakken, 2020; Haralambous et al., 2014; Nielsen et al., 2020; Willis et al., 2016). Some included studies focused on caregivers of persons with dementia who identified delayed dementia diagnosis, often due to beliefs or stigma surrounding dementia, as a barrier to accessing knowledge about dementia-specific services (Baghirathan et al., 2020; Biswas et al., 2022; Gelman, 2010; Ketchum et al., 2022; Krishnamurthi et al., 2022; Nielsen et al., 2020; Shanley et al., 2012).

### Accessing Services

Accessing services was the second stage where CLD caregivers could face facilitators or barriers to accessing and using HCBS. Study results pertaining to accessing services are discussed according to willingness to access services, that is, caregiver or care recipients’ desire or inclination to access services, and caregiver’s ability to access services.

#### Willingness to Access Services

Once caregivers knew about available HCBS, whether they accessed HCBS depended in part on their willingness to do so. The included studies reported facilitators of willingness to access HCBS as referrals from trusted healthcare providers, family, or friends (Horsfall et al., 2016; Ketchum et al., 2022; Koehn et al., 2019), cultural beliefs (Blix & Munkejord, 2022; Motta-Ochoa et al., 2021), availability of culturally diverse or tailored services (Biswas et al., 2022; Browne et al., 2014; Horsfall et al., 2016; Willis et al., 2016; Xiao et al., 2015), culturally acceptable supports (Arora et al., 2020), and caregiving crises (Benedetti et al., 2013; Willis et al., 2016). The reported barriers were beliefs of caregivers, care recipients, and their community about caregiving and dementia (Armstrong et al., 2022; Arora et al., 2020; Benedetti et al., 2013; Berdai Chaouni & De Donder, 2019; Biswas et al., 2022; Browne & Braun, 2017; Casado et al., 2015; Casado & Lee, 2012; Crist & Speaks, 2011; Czapka & Sagbakken, 2020; Giuntoli & Cattan, 2012; Greenwood et al., 2016; Haralambous et al., 2014; Herat-Gunaratne et al., 2020; Horsfall et al., 2016; Ketchum et al., 2022; Koehn et al., 2019; Lee & Smith, 2012; Liu & McDaniel, 2015; Martinez et al., 2022; Martinez & Acosta Gonzalez, 2022; Motta-Ochoa et al., 2021; Nielsen et al., 2020; Nkimbeng et al., 2022; Pound & Greenwood, 2016; Shanley et al., 2012; Strudwick & Morris, 2010; Sun et al., 2014; Williams et al., 2021; Willis et al., 2016; Xiao et al., 2015), and previous negative individual or collective experiences with health and social services (Baghirathan et al., 2020; Berdai Chaouni et al., 2020; Blix & Munkejord, 2022; Giuntoli & Cattan, 2012; Nielsen et al., 2020; Nkimbeng et al., 2022).

Caregivers interviewed in some included studies reported increased willingness to try HCBS recommended by trusted healthcare providers, family, or friends (Horsfall et al., 2016; Ketchum et al., 2022; Koehn et al., 2019). Some caregivers expressed existing or changing cultural beliefs that supported HCBS use (Blix & Munkejord, 2022; Motta-Ochoa et al., 2021). Some caregivers preferred culturally tailored HCBS (Browne et al., 2014; Horsfall et al., 2016; Willis et al., 2016; Xiao et al., 2015) or programs with culturally diverse participants (Biswas et al., 2022), but these were not always accessible due to long waitlists, limited ability of these services to meet care needs, or nonavailability (Biswas et al., 2022; Casado et al., 2015; Krishnamurthi et al., 2022; Liu & McDaniel, 2015; Nielsen et al., 2020; Shanley et al., 2012; Sun et al., 2014; Xiao et al., 2015). Some caregivers were only willing to access HCBS in a crisis (Benedetti et al., 2013; Willis et al., 2016), while others reported that compensation for informal caregiving was more culturally acceptable than HCBS (Arora et al., 2020).

Values around caregiving such as filial piety, defined as cultural norms and values that dictate how children treat their parents, (Bedford & Yeh, 2021), influenced decisions not to access services. Caregivers described the influence of filial piety on caregiving as either positive, for example, a meaningful experience (Armstrong et al., 2022; Arora et al., 2020; Crist & Speaks, 2011; Czapka & Sagbakken, 2020; Herat-Gunaratne et al., 2020; Horsfall et al., 2016; Martinez & Acosta Gonzalez, 2022; Martinez et al., 2022; Motta-Ochoa et al., 2021; Strudwick & Morris, 2010; Williams et al., 2021) or negative, for example, an obligation (Benedetti et al., 2013; Berdai Chaouni & De Donder, 2019; Browne & Braun, 2017; Casado & Lee, 2012; Czapka & Sagbakken, 2020; Herat-Gunaratne et al., 2020; Koehn et al., 2019; Lee & Smith, 2012; Martinez & Acosta Gonzalez, 2022; Nielsen et al., 2020; Shanley et al., 2012; Willis et al., 2016). Caregivers interviewed in some studies believed that the care they provided was superior to professional care due to their dedication and intimate knowledge of the care recipient (Benedetti et al., 2013; Crist & Speaks, 2011; Greenwood et al., 2016; Martinez et al., 2022; Martinez & Acosta Gonzalez, 2022; Nkimbeng et al., 2022; Strudwick & Morris, 2010). Some caregivers feared judgment from their CLD community for using HCBS (Arora et al., 2020; Benedetti et al., 2013; Berdai Chaouni & De Donder, 2019; Czapka & Sagbakken, 2020; Williams et al., 2021), or from the majority population for being perceived as not meeting the expectations of their cultural group or misusing government benefits (Arora et al., 2020; Benedetti et al., 2013; Pound & Greenwood, 2016).

Certain beliefs around dementia decreased willingness to access services. Beliefs that dementia symptoms are part of normal aging or that dementia is sent from God contributed to beliefs that HCBS were not needed (Benedetti et al., 2013; Biswas et al., 2022; Czapka & Sagbakken, 2020; Martinez et al., 2022). Stigma surrounding dementia within some CLD communities led some caregivers to avoid HCBS to conceal the care recipient’s dementia diagnosis (Biswas et al., 2022; Nielsen et al., 2020; Nkimbeng et al., 2022; Sun et al., 2014; Williams et al., 2021; Xiao et al., 2015).

Caregivers participating in some included studies stated they did not use HCBS because the care recipient would have refused them (Arora et al., 2020; Casado et al., 2015; Casado & Lee, 2012; Herat-Gunaratne et al., 2020; Ketchum et al., 2022; Liu & McDaniel, 2015). Reasons for refusal included discomfort with a stranger being in their home (Arora et al., 2020; Crist & Speaks, 2011; Giuntoli & Cattan, 2012; Haralambous et al., 2014; Liu & McDaniel, 2015; Shanley et al., 2012), or preferring family care (Herat-Gunaratne et al., 2020; Ketchum et al., 2022; Strudwick & Morris, 2010).

Negative individual or community experiences with health or social services could decrease willingness to access HCBS. Collective experiences of a CLD community could fuel distrust of services (Baghirathan et al., 2020; Blix & Munkejord, 2022; Giuntoli & Cattan, 2012; Nielsen et al., 2020; Nkimbeng et al., 2022). For example, some Indigenous Sami older adults feared reliving traumas of colonization, so their caregivers hesitated to use HCBS when providers were not Sami and lacked familiarity with their culture (Blix & Munkejord, 2022). Some caregivers felt vulnerable entering what they viewed as mostly all-White community service spaces (Baghirathan et al., 2020). Personal negative experiences, including culturally inappropriate care, also discouraged caregivers from accessing HCBS (Benedetti et al., 2013; Berdai Chaouni et al., 2020; Nielsen et al., 2020).

#### Ability to Access Services

Caregivers who were willing to access HCBS subsequently needed the ability to access HCBS. Included studies reported that staff who were culturally sensitive, culturally or linguistically matched, and/or used interpreters (Browne & Braun, 2017; Haralambous et al., 2014; Ketchum et al., 2022), and assistance from family or friends (Browne et al., 2014; Haralambous et al., 2014) could facilitate access to HCBS. Barriers to accessing HCBS were identified as complex healthcare systems (Armstrong et al., 2022; Benedetti et al., 2013; Casado & Lee, 2012; Haralambous et al., 2014; Horsfall et al., 2016; Ketchum et al., 2022; Martinez et al., 2022; Pound & Greenwood, 2016; Richardson et al., 2019; Xiao et al., 2015) and insurance processes (Browne et al., 2014; Casado et al., 2015; Jung Kim et al., 2019), immigration status (Gelman, 2010; Nkimbeng et al., 2022), language and communication barriers (Czapka & Sagbakken, 2020; Gelman, 2010; Haralambous et al., 2014; Koehn et al., 2019; Pound & Greenwood, 2016; Willis et al., 2016; Xiao et al., 2015), and insensitivity and discrimination (Armstrong et al., 2022; Browne et al., 2014; Greenwood et al., 2016; Motta-Ochoa et al., 2021; Willis et al., 2016). Indigenous and Amish communities faced unique barriers to access. Barriers in Indigenous communities were remote locations, poor housing conditions, and barriers to insurance (Browne et al., 2014; Cooper et al., 2020; Habjan et al., 2012), and barriers in Amish communities were community beliefs, electricity, and financing care (Farrar et al., 2018).

Caregivers interviewed in some included studies found HCBS easier to access when staff at access points were knowledgeable about working with CLD clientele, shared their culture or language, or used interpreters (Browne & Braun, 2017; Haralambous et al., 2014; Ketchum et al., 2022). Friends and family could facilitate access to HCBS by overcoming language barriers or system navigation challenges (Browne et al., 2014; Haralambous et al., 2014).

The complexities of navigating healthcare systems and insurance processes were barriers to accessing services for some CLD caregivers. System navigation challenges included not knowing how to access HCBS or where to find help with the process, which could be exacerbated by language barriers (Armstrong et al., 2022; Benedetti et al., 2013; Casado & Lee, 2012; Haralambous et al., 2014; Horsfall et al., 2016; Ketchum et al., 2022; Martinez et al., 2022; Pound & Greenwood, 2016; Richardson et al., 2019; Xiao et al., 2015). Delays and wait times undermined trust in the system (Haralambous et al., 2014; Pound & Greenwood, 2016). Caregivers in the United States could face insurance process barriers that limited the availability of affordable HCBS (Browne et al., 2014; Casado et al., 2015; Jung Kim et al., 2019). Some caregivers caring for undocumented immigrants did not know how to access support without documentation (Gelman, 2010; Nkimbeng et al., 2022).

Language and communication could be a barrier to accessing HCBS. Caregivers interviewed in some included studies reported that language barriers could interfere with various aspects of accessing services, including completing forms or assessments and clearly expressing needs (Czapka & Sagbakken, 2020; Gelman, 2010; Haralambous et al., 2014; Koehn et al., 2019; Martinez et al., 2022; Pound & Greenwood, 2016; Xiao et al., 2015). The jargon of HCBS was unfamiliar to some caregivers, but they perceived that using jargon helped grant access to HCBS (Pound & Greenwood, 2016; Willis et al., 2016). Some caregivers highlighted a lack of linguistic or cultural support with paperwork that limited their ability to access supports that met their needs (Pound & Greenwood, 2016).

Some caregivers experienced insensitivity, disrespect, or discrimination in their attempts to access HCBS. Caregivers could be rejected by staff who believed that caregivers of certain ethnicities would not want to use HCBS (Armstrong et al., 2022; Willis et al., 2016). Some caregivers found the discussion of intimate details during the assessment process to be insensitive and uncomfortable (Browne et al., 2014; Greenwood et al., 2016).

Some studies reported barriers to access that were specific to Indigenous or Amish communities due to historical and present-day contexts. Indigenous caregivers in rural and remote locations noted that they faced challenges with transportation of services and care recipients in and out of the community (Browne et al., 2014; Cooper et al., 2020; Habjan et al., 2012). Some care recipients could not receive homecare services due to the poor conditions of their homes, such as a lack of indoor potable water (Cooper et al., 2020). Indigenous caregivers interviewed by Habjan et al. (2012) reported that their communities faced inadequate funding and human resources, leading to limited care. Some Native Hawai’ian care recipients did not qualify for Medicaid because of historical land titles in the family that did not contribute to their wealth, leaving them without insurance and rendering HCBS unaffordable (Browne et al., 2014). Farrar et al. (2018) interviewed Amish caregivers who shared that their communities faced challenges related to hesitations to interact with those outside their community and a lack of electricity for any equipment needed. Additionally, communities were conflicted about how to finance HCBS, as the prohibitive costs of healthcare and HCBS rendered the traditional use of community funds unsustainable (Farrar et al., 2018).

### Using Services

The final stage where a CLD caregiver could experience facilitators or barriers was in using HCBS. Included studies reported that culturally appropriate care, service providers or program participants with shared culture and/or language, and culturally tailored activities facilitated positive experiences and continued service use (Baghirathan et al., 2020; Biswas et al., 2022; Blix & Munkejord, 2022; Browne et al., 2014; Browne & Braun, 2017; Cooper et al., 2020; Giuntoli & Cattan, 2012; Greenwood et al., 2016; Haralambous et al., 2014; Herat-Gunaratne et al., 2020; Horsfall et al., 2016; Jung Kim et al., 2019; Ketchum et al., 2022; Krishnamurthi et al., 2022; Motta-Ochoa et al., 2021; Nielsen et al., 2020; Pound & Greenwood, 2016; Shanley et al., 2012; Willis et al., 2016; Xiao et al., 2015), while culturally and linguistically inappropriate care and disrespectful care were barriers to acceptable services and continued HCBS use (Baghirathan et al., 2020; Benedetti et al., 2013; Berdai Chaouni et al., 2020; Berdai Chaouni & De Donder, 2019; Biswas et al., 2022; Browne et al., 2014; Casado et al., 2015; Casado & Lee, 2012; Cooper et al., 2020; Czapka & Sagbakken, 2020; Gelman, 2010; Giuntoli & Cattan, 2012; Greenwood et al., 2016; Herat-Gunaratne et al., 2020; Koehn et al., 2019; Liu & McDaniel, 2015; Motta-Ochoa et al., 2021; Nielsen et al., 2020; Nkimbeng et al., 2022; Pound & Greenwood, 2016; Richardson et al., 2019; Shanley et al., 2012; Strudwick & Morris, 2010; Willis et al., 2016).

Caregivers interviewed in included studies reported that service providers who shared the care recipient’s language or culture facilitated positive experiences with HCBS (Blix & Munkejord, 2022; Browne et al., 2014; Browne & Braun, 2017; Cooper et al., 2020; Greenwood et al., 2016; Haralambous et al., 2014; Herat-Gunaratne et al., 2020; Horsfall et al., 2016; Jung Kim et al., 2019; Ketchum et al., 2022; Krishnamurthi et al., 2022; Nielsen et al., 2020; Pound & Greenwood, 2016; Shanley et al., 2012; Xiao et al., 2015). Some caregivers relied on CLD-led organizations for services for this reason (Baghirathan et al., 2020), while others shared concerns about the limited diversity of service providers (Nkimbeng et al., 2022). Shared language and culture facilitated communication and accelerated the building of trust and rapport between service providers and care recipients (Browne et al., 2014; Greenwood et al., 2016; Ketchum et al., 2022; Pound & Greenwood, 2016; Xiao et al., 2015). Sharing a religious background with service providers promoted the following of religious observances (Greenwood et al., 2016; Pound & Greenwood, 2016). Notably, not all caregivers prioritized culturally or linguistically matched service providers, instead favoring providers who performed their job well and services that fit the care recipient’s needs (Browne et al., 2014; Horsfall et al., 2016; Nielsen et al., 2020). Several caregivers highlighted that service providers who understood and respected the care recipient’s culture, built trust, promoted dignity, assessed cultural and religious needs, communicated openly with care recipients and families, engaged in culturally tailored activities, and used interpreters as needed could meet the care recipient’s needs irrespective of the provider’s background (Browne et al., 2014; Giuntoli & Cattan, 2012; Herat-Gunaratne et al., 2020; Motta-Ochoa et al., 2021; Pound & Greenwood, 2016; Willis et al., 2016). Group programs with culturally tailored activities or participants with shared culture and/or language were enjoyable for some care recipients, especially when staff facilitated relationships between participants; however, programs were not always designed for culturally diverse groups (Biswas et al., 2022; Browne & Braun, 2017; Krishnamurthi et al., 2022; Motta-Ochoa et al., 2021).

Conversely, a lack of culturally or linguistically appropriate care resulted in negative experiences for caregivers and care recipients. Language barriers were the most cited challenge in using HCBS (Baghirathan et al., 2020; Benedetti et al., 2013; Berdai Chaouni & De Donder, 2019; Biswas et al., 2022; Casado et al., 2015; Casado & Lee, 2012; Czapka & Sagbakken, 2020; Gelman, 2010; Greenwood et al., 2016; Herat-Gunaratne et al., 2020; Koehn et al., 2019; Liu & McDaniel, 2015; Nielsen et al., 2020; Nkimbeng et al., 2022; Pound & Greenwood, 2016; Richardson et al., 2019; Shanley et al., 2012; Willis et al., 2016). Language barriers limited the care recipient’s ability to express their needs, which could result in suboptimal care provision, caregiver anxiety, and discontinuation of services (Berdai Chaouni & De Donder, 2019; Nielsen et al., 2020; Willis et al., 2016). Some caregivers were not allowed to engage in care activities despite their ability to bridge cultural and linguistic gaps between care recipients and service providers (Blix & Munkejord, 2022). Several caregivers shared that language barriers could prevent care recipients from fully participating in services held in group settings, leading them to feel isolated, abandoned, and unwelcome (Benedetti et al., 2013; Biswas et al., 2022; Koehn et al., 2019; Shanley et al., 2012; Willis et al., 2016). Several caregivers found that language barriers could be exacerbated by cultural differences in non-verbal communication, diagnosis-related communication barriers, or incorrect assumptions that a care recipient understood the service provider (Greenwood et al., 2016; Pound & Greenwood, 2016; Richardson et al., 2019; Shanley et al., 2012). Miscommunication, lack of communication, or misunderstandings about what services the service provider would deliver could negatively affect care and relationships with service providers (Blix & Munkejord, 2022; Cooper et al., 2020; Giuntoli & Cattan, 2012; Herat-Gunaratne et al., 2020).

Food, entertainment, and religion were identified as important aspects of culturally appropriate care. Food provided for care recipients could be unfamiliar and unappetizing, and service providers did not always follow cultural and religious beliefs and traditions around food (Baghirathan et al., 2020; Berdai Chaouni et al., 2020; Berdai Chaouni & De Donder, 2019; Casado et al., 2015; Czapka & Sagbakken, 2020; Herat-Gunaratne et al., 2020; Nielsen et al., 2020; Shanley et al., 2012; Willis et al., 2016). Unfamiliar television, pastimes, or music could isolate the care recipient in group settings (Baghirathan et al., 2020; Nielsen et al., 2020). When service providers did not understand or respect care recipients’ religious needs, such as sex-matched service providers or washing rituals, they often did not meet these needs (Greenwood et al., 2016; Shanley et al., 2012). Factors contributing to unmet religious needs were rushing through services and unpredictable timing of homecare services (Berdai Chaouni et al., 2020; Berdai Chaouni & De Donder, 2019; Greenwood et al., 2016; Nielsen et al., 2020; Shanley et al., 2012).

Caregivers in some included studies found that care recipients received undignified and disrespectful care, which could lead to distrust, frustration, and the cessation of services (Berdai Chaouni et al., 2020; Browne et al., 2014; Nielsen et al., 2020; Pound & Greenwood, 2016; Strudwick & Morris, 2010). Caregivers shared examples of racism, discrimination, cultural insensitivity, and disrespect by service providers, including making assumptions based on ethnicity, not meeting a care recipient’s need for privacy, rushing the care recipient through care routines, and an inability or unwillingness to meet cultural needs (Baghirathan et al., 2020; Berdai Chaouni et al., 2020; Biswas et al., 2022; Browne et al., 2014; Czapka & Sagbakken, 2020; Giuntoli & Cattan, 2012; Haralambous et al., 2014; Motta-Ochoa et al., 2021; Nielsen et al., 2020; Pound & Greenwood, 2016; Strudwick & Morris, 2010).

## Discussion

This scoping review aimed to synthesize the current state of the literature exploring facilitators and barriers to the access and use of HCBS for informal caregivers of CLD older adults. This review was motivated by an increasing need to understand how HCBS can better meet the needs of the growing population of CLD caregivers (Greenwood et al., 2015).

### Overall Findings

Caregivers of CLD older adults faced facilitators and barriers to HCBS at each temporal stage of access and use: knowledge of HCBS, willingness to use HCBS, ability to access HCBS, and use of HCBS. An overarching theme emerging from this review is the need for culturally appropriate care that can adapt to meet the various needs of CLD clients, requiring service providers, organizations, and healthcare systems to commit to changing the status quo of service delivery. Facilitators and barriers identified in this review often related to language, culturally appropriate care, beliefs about caregiving and dementia, trust, discrimination, system navigation, informal networks, and the diversity of service providers. Notably, included studies present far more barriers than facilitators, which may reflect both the extent of challenges faced by CLD caregivers and increasing recognition of these challenges in the literature. Only four included studies reported on facilitators to CLD caregivers’ ability to access HCBS, the least citations for any category of facilitator or barrier. This underrepresentation could be reflective of a true lack of facilitators for the ability to access services, a gap in the literature investigating and reporting on these facilitators, or an indication that facilitators may not be directly linked to cultural or linguistic differences.

Existing literature echoes facilitators to HCBS for CLD caregivers as a knowledge gap (Greenwood et al., 2015; Kenning et al., 2017); however, our review contributes more context around facilitators to address this gap. This review adds the role of family and friends, desperation and caregiver crises, immigrant-serving organizations, and trusting relationships with healthcare providers in facilitating CLD caregivers’ access and use of HCBS. Findings supported by the current literature include the mistrust of HCBS, limited availability of highly desired culturally tailored services, influence of filial piety on care decisions, and experiences with cultural insensitivity and racism while interacting with HCBS (Duran-Kiraç et al., 2021; Greenwood et al., 2015; Johl et al., 2016; Kenning et al., 2017). Existing literature highlights strong preferences for culturally and linguistically matched care providers (Greenwood et al., 2015; Kenning et al., 2017), while our review adds the viewpoint that skilled, respectful, and culturally sensitive care providers are acceptable to some CLD caregivers.

### Implications for Research

Future research should continue exploring the experiences of CLD populations with HCBS access and use to inform healthcare policies, education, and practice. Studies included in the review emphasize the importance of exploring the differences and similarities within and between groups and the experiences of caregivers of various ethnicities, socioeconomic statuses, genders, and acculturation levels (Arora et al., 2020; Berdai Chaouni & De Donder, 2019; Browne & Braun, 2017; Czapka & Sagbakken, 2020; Farrar et al., 2018; Liu & McDaniel, 2015; Richardson et al., 2019). Future research could address barriers at each temporal stage of accessing and using HCBS, focusing on disseminating knowledge, developing HCBS that are accessible and acceptable to CLD populations, and addressing language barriers. The literature could be reviewed further to capture perspectives of service providers and care recipients or to capture suggestions from stakeholders on how to improve the accessibility and acceptability of HCBS.

### Implications for Practice

Review findings suggest that healthcare and service providers can better support CLD clients by working within a cultural humility framework, avoiding assumptions based on cultural background, and completing thorough assessments of clients’ needs. The concept of cultural humility embodies critical self-reflection, humility, lifelong learning, and recognition of power imbalances to promote empowerment, respect, and optimal care that is culturally appropriate for clientele from all backgrounds (Foronda et al., 2016). Included studies emphasized that providers should not make assumptions about CLD caregivers’ and care recipients’ willingness or need for HCBS (Arora et al., 2020; Gelman, 2010; Strudwick & Morris, 2010). Instead, providers should explore individual, family, and cultural needs and values and present options for HCBS accordingly (Giuntoli & Cattan, 2012; Greenwood et al., 2016; Haralambous et al., 2014; Richardson et al., 2019; Xiao et al., 2015), which may improve reception to HCBS (Casado et al., 2015; Lee & Smith, 2012). Review findings promote assessing for cultural and religious needs to be able to better understand, respect, and meet clients’ needs. The review identified family and friends as important facilitators to knowledge and ability to access HCBS. Healthcare and service providers should therefore assess caregiver access to support from family and friends and supplement support as needed. Review results highlight crises or desperation as motivators to accessing and using HCBS. Healthcare providers may consider developing plans with families before a crisis occurs to establish adequate support in the event of a caregiving crisis.

### Implications for Policy

Review results highlight the need for policy changes to improve HCBS. System-level policy changes must target disparities within healthcare systems, system navigation, and increased CLD representation at all levels of healthcare delivery and policy. Systems in Westernized countries are designed for their majority populations and disproportionately disadvantage CLD populations (Bell, 2021). Policies and systems need to be redesigned to meet the needs of a diverse population. Studies included in the review advocate for seeking out and listening to perspectives of CLD caregivers to inform policies to develop affordable, accessible, and acceptable HCBS for CLD communities (Berdai Chaouni & De Donder, 2019; Berdai Chaouni et al., 2020; Blix & Munkejord, 2022; Browne & Braun, 2017; Browne et al., 2014; Casado & Lee, 2012; Farrar et al., 2018; Sun et al., 2014). Funding for HCBS should consider the needs of CLD caregivers, for example, allocating funding for culturally tailored services, compensation for informal caregivers in lieu of HCBS, and changes to HCBS needed to better support CLD clients. Review findings identified that system navigation is challenging for many CLD caregivers, and interventions to facilitate system navigation may improve access to HCBS (Ketchum et al., 2022; Liu & McDaniel, 2015; Pound & Greenwood, 2016; Shanley et al., 2012; Willis et al., 2016). Policies should address the complexities of the healthcare system and ensure system navigation supports are available. Review findings promote increased cultural and linguistic diversity in the healthcare field (Ketchum et al., 2022; Lee & Smith, 2012; Sun et al., 2014), which can improve a healthcare system’s ability to provide safe and appropriate care to CLD populations (Bell, 2021). Changes towards anti-racist health program pedagogy may better support CLD students and facilitate increased diversity in healthcare (Bell, 2021).

Policy changes at HCBS organizations should address language barriers, pairing CLD clients with preferred service providers, access to culturally appropriate and tailored services, and system navigation. Included studies encourage the availability and use of strategies and tools to overcome language barriers, including language-matching service providers, interpreter services, simplified language, engaging caregivers in activities when possible, and building relationships with CLD-led organizations to promote culturally and linguistically appropriate dissemination of information (Blix & Munkejord, 2022; Casado et al., 2015; Casado & Lee, 2012; Koehn et al., 2019; Lee & Smith, 2012; Willis et al., 2016; Xiao et al., 2015). Review findings promote pairing CLD clients with services providers who share their cultural background or who provide culturally appropriate care. Findings indicate that culturally tailored services are highly desired by many CLD caregivers but are not always available or accessible. Included studies suggest including culturally inclusive activities into programming (Biswas et al., 2022) and developing partnerships between mainstream and ethno-specific services that could improve the availability and quality of both types of services (Casado et al., 2015; Koehn et al., 2019). HCBS organizations could also facilitate support with system navigation, for example, by connecting new and experienced caregivers (Pound & Greenwood, 2016).

### Strengths and Potential Limitations

Strengths of this scoping review include the representation of a broad range of perspectives through the inclusion of publications that represent numerous CLD identities in nine countries without limitations related to diagnoses. This review also has limitations. The search strategy was limited to five databases and by publication date, English language, and peer-reviewed publications. Inclusion of articles that were represented by gray literature and journals in non-English languages may have strengthened the review particularly given the study focus on CLD caregivers. Our focus on Westernized countries limits the generalizability of findings to these countries. Only facilitators and barriers with clear ties to culture and language were extracted, so relevant factors may have been overlooked. Conversely, findings may be overly attributed to culture since most included studies did not compare CLD and majority population experiences. Lastly, included studies ranged from poor to high quality, which may affect review results. However, the use of a single appraiser and insufficient methodological data reported in some included articles limited the quality of the critical appraisal.

## Conclusion

This scoping review sought to review the empirical literature on CLD informal caregivers to identify facilitators and barriers to their access and use of HCBS in Westernized countries. Review findings indicate that while some caregivers from CLD communities may prefer to care for their loved ones without formal services, many CLD caregivers want and can benefit from HCBS that provide culturally appropriate care. Barriers to HCBS access and use are extensive, and stakeholders must work to improve the accessibility and acceptability of HCBS for CLD caregivers. Going forwards, stakeholders must seek to engage CLD community members in research, policy, and HCBS development and implementation to help ensure that this population’s needs are understood and met with appropriate services.

## Supplemental Material

Supplemental Material - Access and Use of Services by Caregivers of Older Adults: A Scoping Review of Cultural and Linguistic Diversity Click here for additional data file. Supplemental Material for Access and Use of Services by Caregivers of Older Adults: A Scoping Review of Cultural and Linguistic Diversity by Danielle Knipping, BScN, Anna Garnett, PhD, and Bingfang Bianca Jiang, BScN in Journal of Applied Gerontology
